# Supplementary material for: Massively parallel reporter assays and mouse transgenic assays provide correlated and complementary information about neuronal enhancer activity
Source: Nat Commun. 2025 May 23;16:4786. doi: 10.1038/s41467-025-60064-1 (PMC12098896; doi:10.1038/s41467-025-60064-1)
Supplement: Supplementary file 1 — Supplementary Information [file 41467_2025_60064_MOESM1_ESM.pdf]

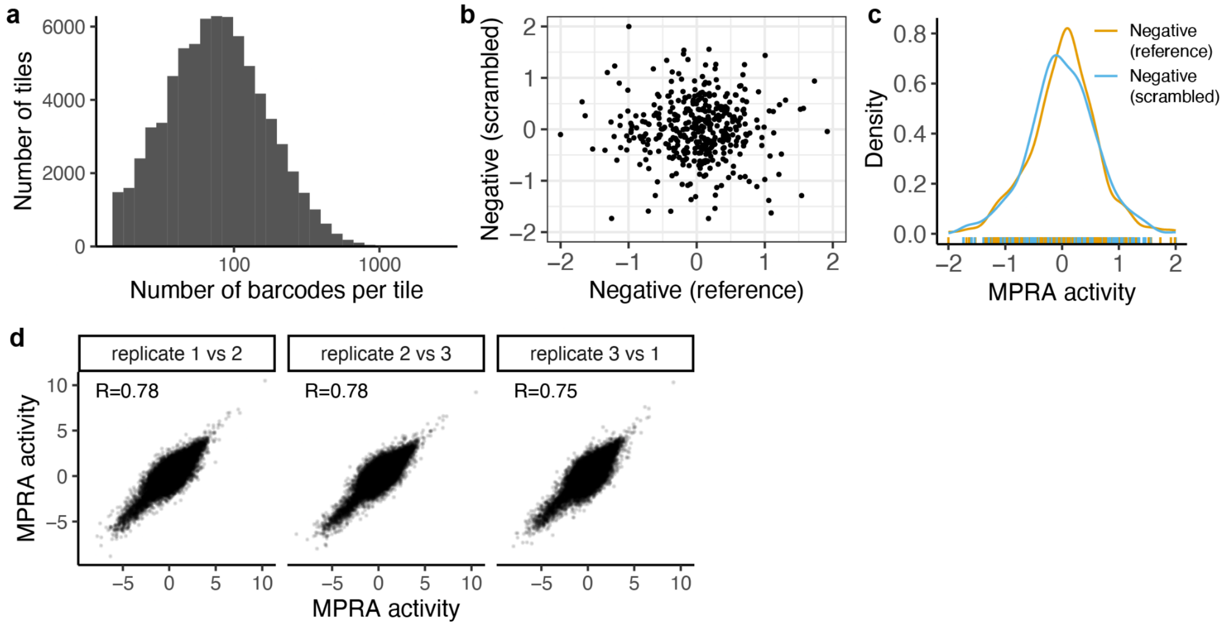

Supplementary Figure 1. (a) Histogram of the number of barcodes per tile. (b) Scatterplot of MPRA activity comparing genomic reference negative elements and their dinucleotide scrambled equivalents. N=454. (c) Density plot of MPRA activity of genomic reference negative elements and their dinucleotide scrambled equivalents. Rug plot below indicates individual observations. (d) Correlation of MPRA activity between biological replicates. R - Pearson correlation. N=73,367.

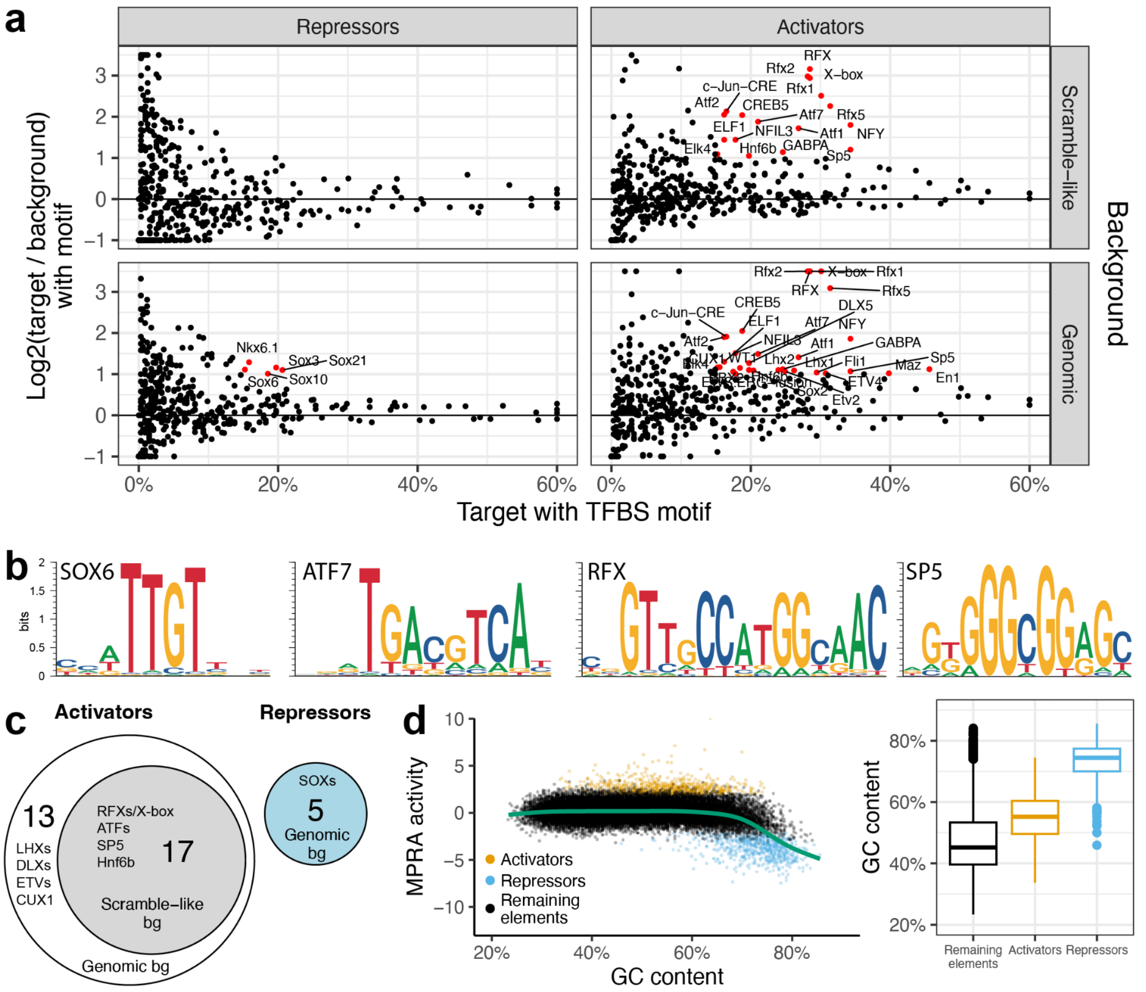

Supplementary Figure 2. **Neuronal WTC11 MPRA results validation. (a)** TFBS (transcription factor binding site) enrichment in enhancer activator (N = 309) or repressor (N =335) tiles compared to enhancer elements with scramble negative levels of activity (N = 20,974, "scramble-like") or genomic background elements (N = 50,000; "genomic"). Log2-fold change was curbed at -1 and 3.5. Fraction of tiles with motif was curbed at 60%. Only TFBSs present in more than 15% of targets, with two times increase in presence from background to target set (corresponding to log2(2) = 1 cutoff) and FDR<1% are labeled. Top-right panel is the same as Figure 2b. **(b)** Examples of TFBSs enriched in enhancer activator tiles compared to enhancer elements with scramble negative levels of activity. **(c)** Overlap between TFBS enriched in different analyses from previous panel. TFs with similar names collapsed (e.g. "RFXs"). **(d)** Relationship between MPRA activity and GC content. Left: scatterplot. Green line is a smooth mean generated by a general additive model with restricted maximum likelihood parameter selection. Right: boxplot of GC content binned by tile category (activators N = 742, repressors N = 732, remaining elements N = 48,609). Hinges of boxplots span interquartile range (IQR), line in the middle is median, thickness (height) is proportional to number of overlapping tiles. Whiskers extend from the hinge to the largest value no further than 1.5 * IQR from the hinge, data points exceeding this range are plotted.

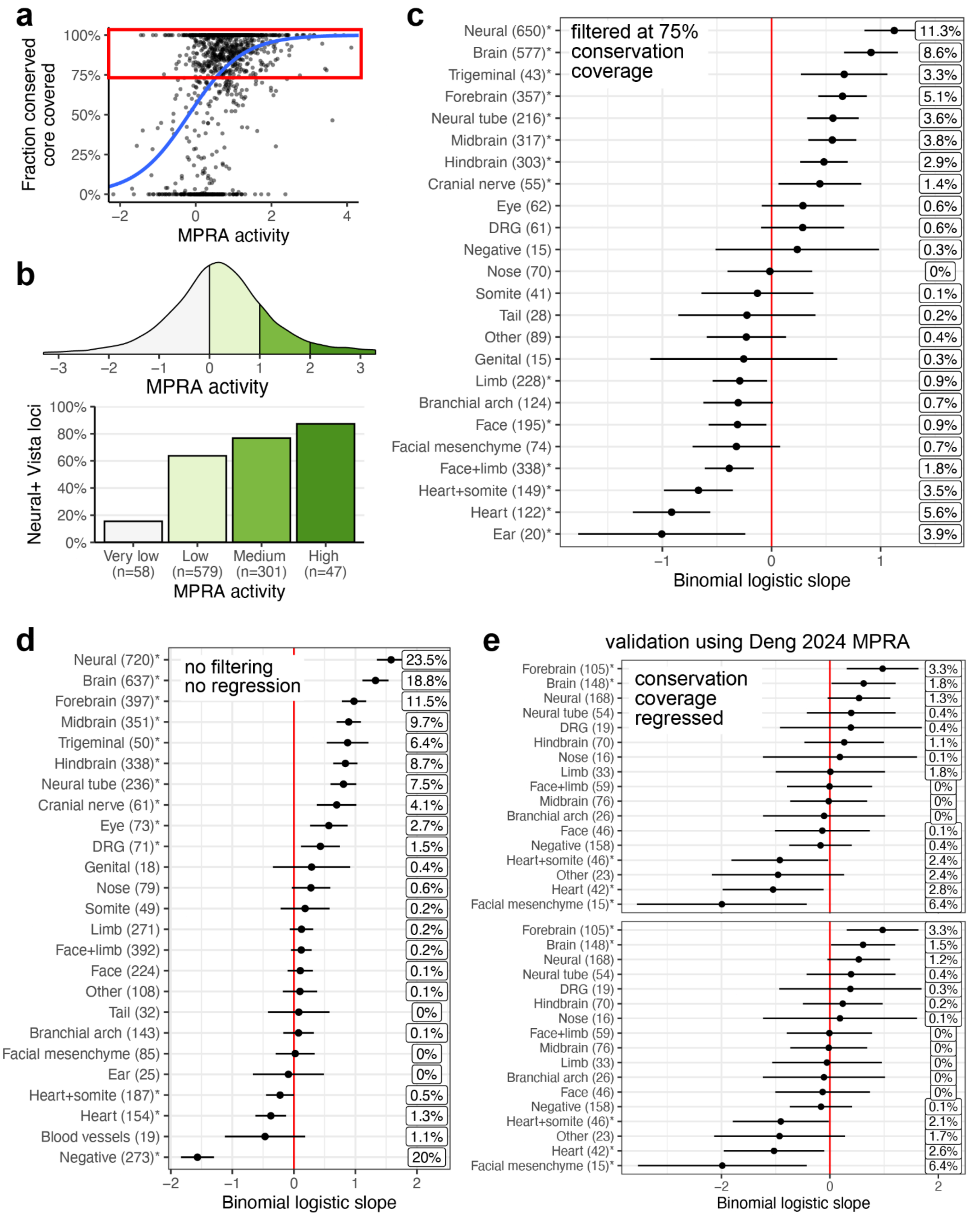

Supplementary Figure 3. **Predicting transgenic assay activity using a MPRA-based, coverage-marginalized model. (a)** Relationship between fraction of conserved core covered and MPRA activity. The blue line is the binomial-link GLM regression on this variable. Instead of including this variable as covariate in GLM, VISTA elements with coverage lower than 75% were removed prior to modeling. **(b)** Alternative visualization of relationship between MPRA activity and transgenic assay

activity. Top: MPRA activity bins. Bottom: fraction of neural-positive VISTA loci by MPRA activity bin. Numbers below bars are counts of VISTA elements. Only "well-covered" elements included, as defined above (N = 985). **(c)** Results of the GLM predicting binomial transgenic assay activity of well-covered VISTA elements from MPRA activity. (**d**) Results of the GLM, without filtering or regression for conservation coverage. (e) Validation of the GLM approach using the Deng 2024 MPRA[50] in primarily cortical cells. Asterisks indicate p-value < 0.05 (likelihood ratio test, no multiple testing correction). Boxed percentages to the right are Nagelkerke R^2 measures. Bars extend two standard errors of the mean in each direction. DRG = dorsal root ganglia. Cranial nerves category does not include the trigeminal nerve, as per VISTA Browser.

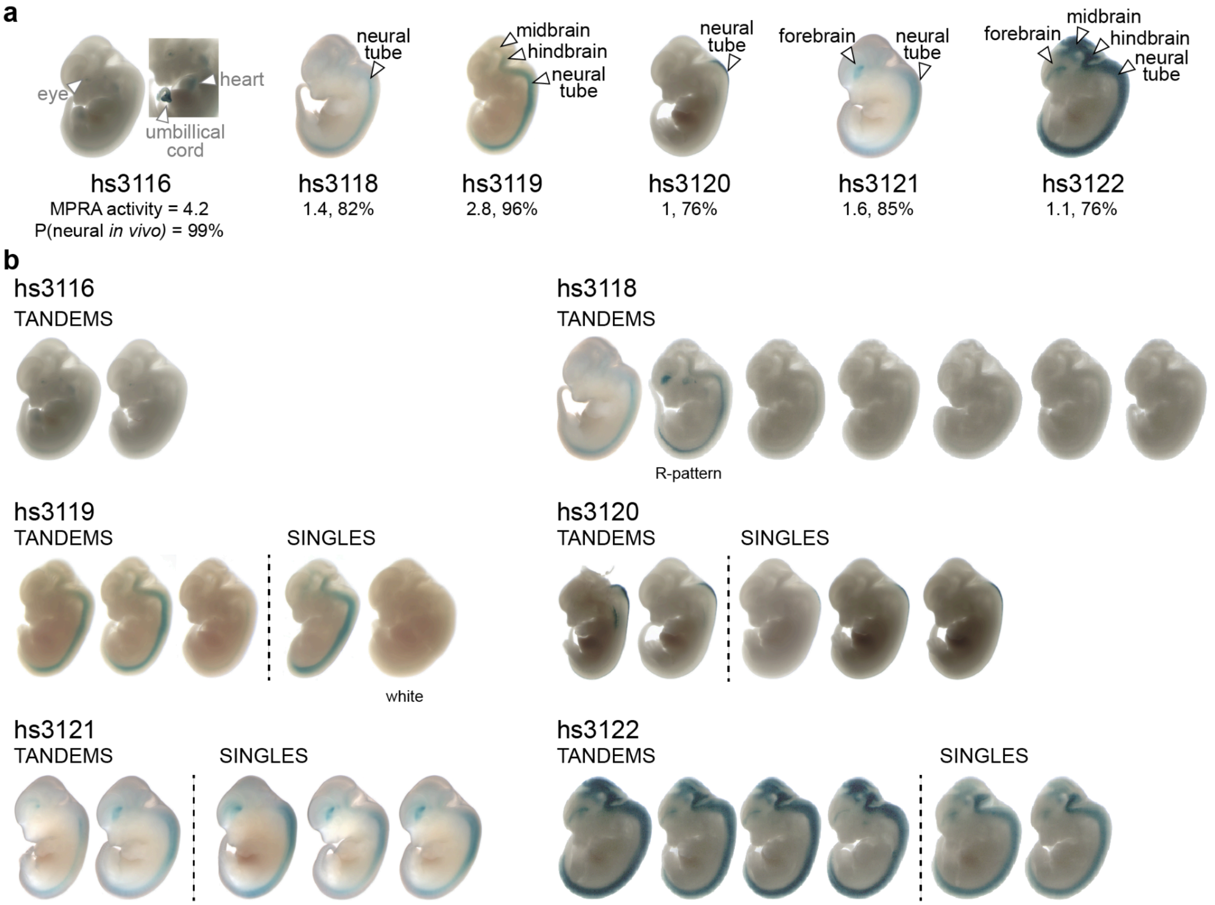

Supplementary Figure 4. Experimental validation of the model predicting transgenic reporter activity from MPRA activity. (a) Representative stained embryos. All elements showed reproducible reporter activity, with 5 out of 6 (83%) having reproducible activity in neural tissues. White arrowheads indicate structures with reproducible reporter activity. Gray text indicates non-neural tissues. MPRA activity of the overlapping tile with highest activity, as well as the GLM-predicted likelihood of this element being positive for neural activity *in vivo,* are indicated under each element. **(b)** All transgenic embryos. Tandems = embryos that were genotyped as positive for reporter integration at the safe harbor locus and presence of the plasmid backbone indicating higher transgene copy number with strong, reproducible pattern. Singles = embryos that were genotyped positive for reporter integration at the safe harbor locus and negative for plasmid backbone, indicating lower transgene copy number with weaker, but reproducible pattern. R-pattern - embryos with deviant pattern indicative of random (R) genomic insertion. Embryo without any staining is labeled "white".

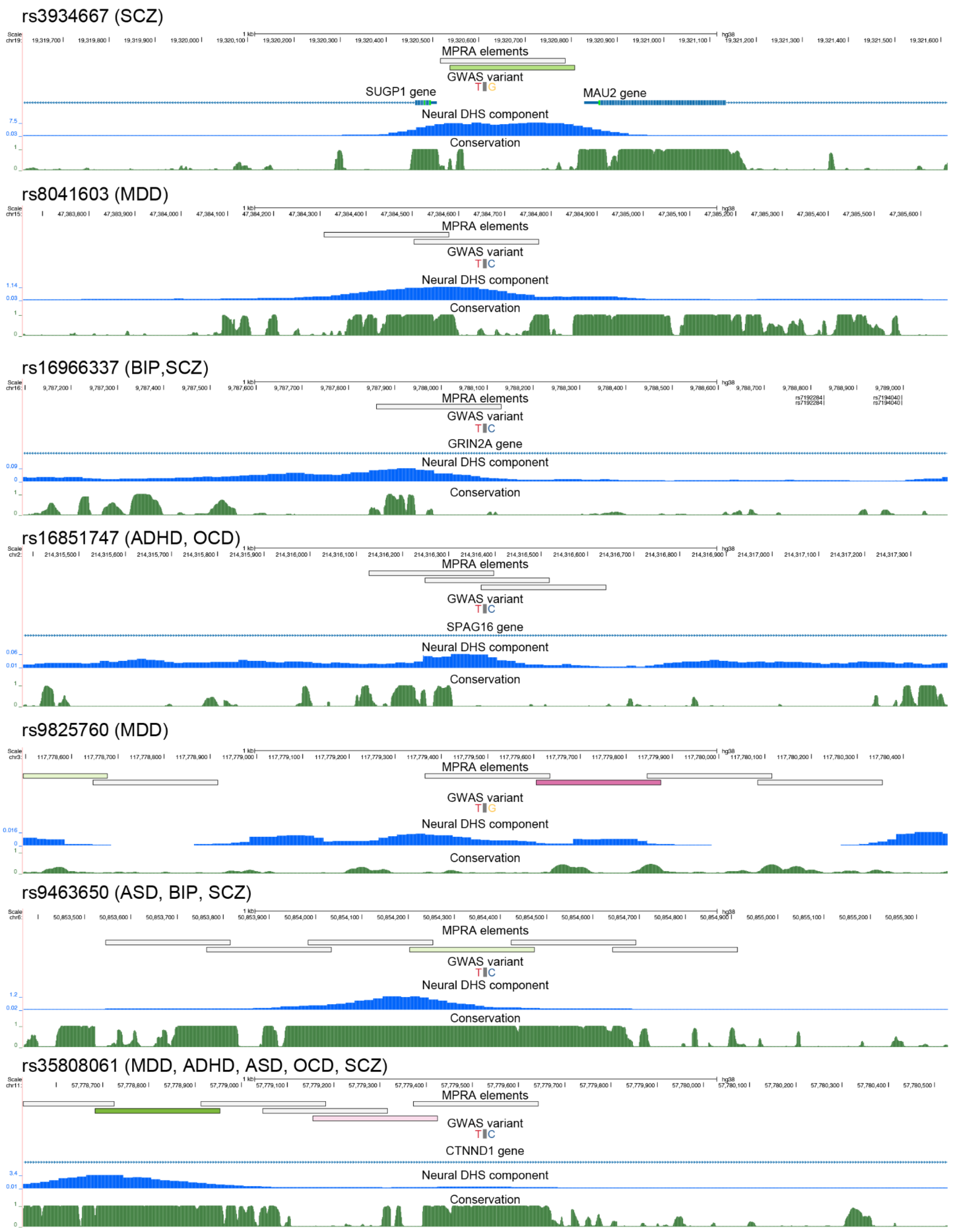

Supplementary Figure 5. **Genomic tracks of the seven significant GWAS variants.** Conservation is shown as the PhastCons UCSC track for 30 mammals (27 primates). MPRA elements are colored by their MPRA activity, see inset. Neural DHS component from Meuleman 2020[33]. MDD = major depressive disorder, BIP = bipolar disorder, SCZ = schizophrenia, ASD = autism spectrum disorder.

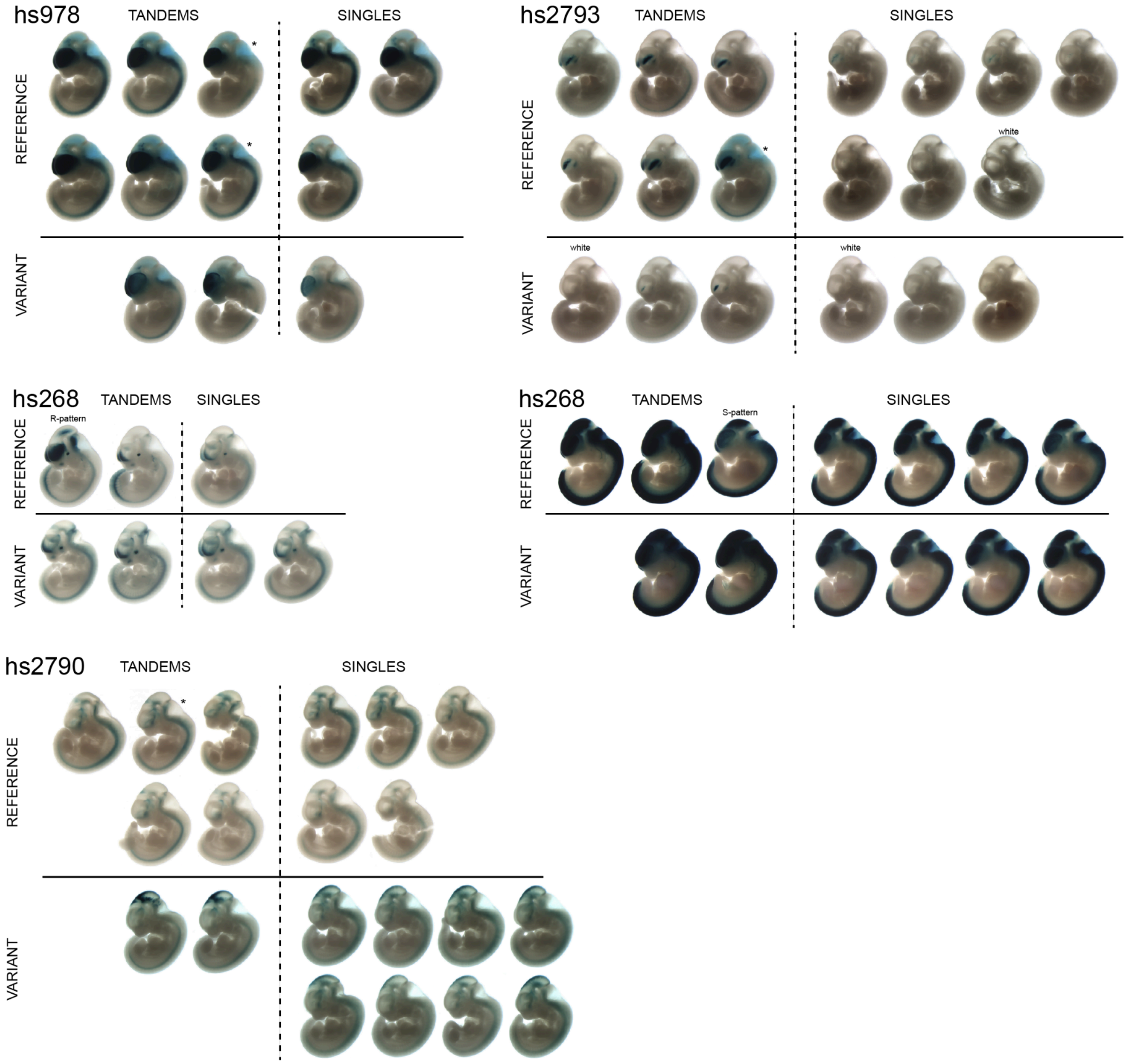

Supplementary Figure 6. Results of transgenic mouse assay. Tandems = embryos that were genotyped as positive for reporter integration at the safe harbor locus and presence of the plasmid backbone indicating higher transgene copy number with strong, reproducible pattern. Singles = embryos that were genotyped positive for reporter integration at the safe harbor locus and negative for plasmid backbone, indicating lower transgene copy number with weaker, but reproducible pattern. Asterisks - embryos with uncertain genotype. R-pattern - embryos with deviant pattern indicative of random (R) genomic insertion. S-pattern - tandem embryos with expression pattern resembling that of single-genotyped embryos. Embryos without any staining are labeled "white".

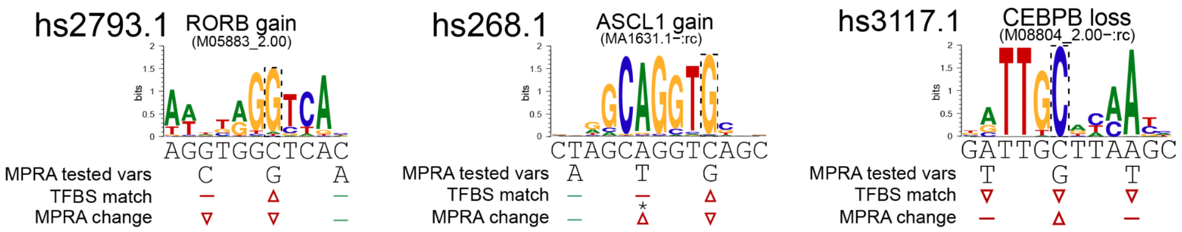

Supplementary Figure 7. TFBSs (transcription factor binding sites) predicted to be affected by the outlined variants, but inconsistent with MPRA effects of other TFBS overlapping variants. TFBS and MPRA change symbols are colored green if matching and red if not. Arrowheads indicate an increase or decrease, flat line indicates no effect (assuming predicted TFBSs are activating).
