# Supplementary material for: Massively parallel reporter assays and mouse transgenic assays provide correlated and complementary information about neuronal enhancer activity
Source: Nat Commun. 2025 May 23;16:4786. doi: 10.1038/s41467-025-60064-1 (PMC12098896; doi:10.1038/s41467-025-60064-1)
Supplement: Supplementary file 2 — Description of Additional Supplementary Information [file 41467_2025_60064_MOESM2_ESM.docx]

**Supplementary Data 1. Results of categorical enrichment analysis.** P-values are FDR-corrected and significant at -log10(0.05) = 1.3 (all, excluding lncRNA exon category). Two-sided Mann-Whitney test was conducted on ranks of elements in a given category vs ranks of scramble negative controls. N = number of tiles overlapping a category.

**Supplementary Data 2. Raw results of HOMER run. Known motifs.** Motif enrichment p-value is from a binomial test.

**Supplementary Data 3. Raw results of HOMER run. De novo motifs.** Motif enrichment p-value is from a binomial test.

**Supplementary Data 4. Results of epigenomic enrichment analysis.** Median_difference_5k is median MPRA activity difference between top 5,000 by epigenomic signal and remaining enhancer tiles. Rank_5k is median_difference_5k ranked.

**Supplementary Data 5. Significant GWAS variants.** Probability that an element overlapping the variant is neural-positive in vivo is the maximum probability obtained by applying the neural model from Figure 3 to the reference tile and reference tile with the variant. Putative neuronal target genes were identified by nearest gene method (MAU2, SEMA6D, SPAG16, TFAP2B and TFAP2D) or using Activity-By-Contact (ABC) model trained on WTC11 neurons or prenatal week 18 prefrontal cortex neurons (CTNDD1, GRIN2A).

**Supplementary Data 6. Sources for data and software used in this study.**

**Supplementary Data 7. Number of peaks/tiles covered in the final library.** Numbers are not mutually exclusive.
