# Supplementary material for: Massively parallel reporter assays and mouse transgenic assays provide correlated and complementary information about neuronal enhancer activity
Source: Nat Commun. 2025 May 23;16:4786. doi: 10.1038/s41467-025-60064-1 (PMC12098896; doi:10.1038/s41467-025-60064-1)
Supplement: Supplementary file 4 — Reporting Summary [file 41467_2025_60064_MOESM4_ESM.pdf]

nature portfolio

Corresponding author(s): Nadav Ahituv

Last updated by author(s): May 2, 2025

Reporting Summary

Nature Portfolio wishes to improve the reproducibility of the work that we publish. This form provides structure for consistency and transparency in reporting. For further information on Nature Portfolio policies, see our Editorial Policies and the Editorial Policy Checklist.

Statistics

For all statistical analyses, confirm that the following items are present in the figure legend, table legend, main text, or Methods section.

n/a	Confirmed	
☐	☒	The exact sample size (*n*) for each experimental group/condition, given as a discrete number and unit of measurement
☐	☒	A statement on whether measurements were taken from distinct samples or whether the same sample was measured repeatedly
☐	☒	The statistical test(s) used AND whether they are one- or two-sided<br>*Only common tests should be described solely by name; describe more complex techniques in the Methods section.*
☐	☒	A description of all covariates tested
☐	☒	A description of any assumptions or corrections, such as tests of normality and adjustment for multiple comparisons
☐	☒	A full description of the statistical parameters including central tendency (e.g. means) or other basic estimates (e.g. regression coefficient) AND variation (e.g. standard deviation) or associated estimates of uncertainty (e.g. confidence intervals)
☐	☒	For null hypothesis testing, the test statistic (e.g. *F*, *t*, *r*) with confidence intervals, effect sizes, degrees of freedom and *P* value noted<br>*Give P values as exact values whenever suitable.*
☒	☐	For Bayesian analysis, information on the choice of priors and Markov chain Monte Carlo settings
☒	☐	For hierarchical and complex designs, identification of the appropriate level for tests and full reporting of outcomes
☐	☒	Estimates of effect sizes (e.g. Cohen's *d*, Pearson's *r*), indicating how they were calculated

Our web collection on statistics for biologists contains articles on many of the points above.

Software and code

Policy information about availability of computer code

Data collection	Illumina NextSeq 500 software Sep 2022.
Data analysis	R 4.3.2 with tidyverse 2.0.0, ggrastr 1.0.2, ggplot2 3.5.060 and ggrepel 0.9.561, rms 6.8-0, motifbreakR 2.16.0. and MPRAflow 2.3.4. HOMER 4.11, bedtools 2.31.1, ENCODE atac-seq-pipeline 2.2.1, ENCODE chip-seq-pipeline 2.2.1, samtools 1.10, Bowtie2 2.3.5.1, MarkDuplicates 2.26.0, ABC 1.0.0, BCalm 0.99.0

For manuscripts utilizing custom algorithms or software that are central to the research but not yet described in published literature, software must be made available to editors and reviewers. We strongly encourage code deposition in a community repository (e.g. GitHub). See the Nature Portfolio guidelines for submitting code & software for further information.

Data

Policy information about availability of data

All manuscripts must include a data availability statement. This statement should provide the following information, where applicable:

- Accession codes, unique identifiers, or web links for publicly available datasets
- A description of any restrictions on data availability
- For clinical datasets or third party data, please ensure that the statement adheres to our policy

All raw and processed data relating to the MPRA experiment can be found on the ENCODE portal under ENCSR865OZI, ENCSR517VUU, ENCSR548AQS and ENCSR257CZP. Data related to transgenic assays can be found at VISTA Enhancer Browser (enhancer.lbl.gov).

Research involving human participants, their data, or biological material

Policy information about studies with human participants or human data. See also policy information about sex, gender (identity/presentation), and sexual orientation and race, ethnicity and racism.

Reporting on sex and gender	not relevant
Reporting on race, ethnicity, or other socially relevant groupings	not relevant
Population characteristics	not relevant
Recruitment	not relevant
Ethics oversight	not relevant

Note that full information on the approval of the study protocol must also be provided in the manuscript.

Field-specific reporting

Please select the one below that is the best fit for your research. If you are not sure, read the appropriate sections before making your selection.

[x] Life sciences [ ] Behavioural & social sciences [ ] Ecological, evolutionary & environmental sciences

For a reference copy of the document with all sections, see nature.com/documents/nr-reporting-summary-flat.pdf

Life sciences study design

All studies must disclose on these points even when the disclosure is negative.

Sample size	No sample size calculations were performed. For MPRA we did the standard triplicate. For transgenic assays we aimed at having a minimum of two tandem-genotype embryos, which is our standard for reporting a reproducibly positive or negative element.
Data exclusions	For MPRA, we did not exclude any data. For transgenic assays, only embryos that were genotyped as having a reporter insertion at the targeted locus are shown.
Replication	For MPRA, correlation between replicates is reported. For transgenic assays all correctly targeted embryos results are included.
Randomization	No randomization was performed. Experiments followed a well-established protocol which yields reproducible results well within the margin of error of the reported effect sizes.
Blinding	No blinding was performed. The impact of variants on transgenic assay in this manuscript was clear, and all data is included for the readers and reviewers to assess.

Reporting for specific materials, systems and methods

We require information from authors about some types of materials, experimental systems and methods used in many studies. Here, indicate whether each material, system or method listed is relevant to your study. If you are not sure if a list item applies to your research, read the appropriate section before selecting a response.

Materials & experimental systems

n/a	Involved in the study	
x		Antibodies
	x	Eukaryotic cell lines
x		Palaeontology and archaeology
	x	Animals and other organisms
x		Clinical data
x		Dual use research of concern
x		Plants

Methods

n/a	Involved in the study	
x		ChIP-seq
x		Flow cytometry
x		MRI-based neuroimaging

Eukaryotic cell lines

Policy information about cell lines and Sex and Gender in Research

Cell line source(s)	Differentiated human excitatory neurons were derived from a male hiPSCs in the WTC11 background where a doxycycline-inducible neurogenin 2 transgene was integrated into the AAVS1 locus (PMID: 28966121). This cell line was generated by Dr. Li Gan's lab at the Gladstone Institutes from the parental WTC11 line. The WTC11 cell line was generated with donor consent at the Gladstone Institutes by Dr. Bruce Conklin's laboratory. We obtained the cells from the lab of Dr. Yin Shen at the University of California, San Francisco (UCSF).
Authentication	Line produced neurons upon doxycyclin treatment.
Mycoplasma contamination	All cell lines in our lab are routinely tested for mycoplasma contamination.
Commonly misidentified lines (See ICLAC register)	No commonly misidentified cells were used.

Animals and other research organisms

Policy information about studies involving animals; ARRIVE guidelines recommended for reporting animal research, and Sex and Gender in Research

Laboratory animals	Transgenic mouse assays were performed in Mus musculus FVB strain mice at embryonic day E11.5.
Wild animals	not relevant
Reporting on sex	Animals of both sexes were used for analysis. We observed no variability consistent with potential sex-specific expression of the enhancers in questions.
Field-collected samples	not relevant
Ethics oversight	All animal work was reviewed and approved by the Lawrence Berkeley National Laboratory Animal Welfare and Research Committee. Animals were inspected weekly by the Chair of the Animal Welfare and Research Committee and the head of the animal facility in consultation with the veterinary staff. The LBNL ACF is accredited by the American Association for the Accreditation of Laboratory Animal Care International (AAALAC).

Note that full information on the approval of the study protocol must also be provided in the manuscript.

Plants

Seed stocks	not relevant
Novel plant genotypes	not relevant
Authentication	not relevant
